# Prevalence of multimorbidity and associated treatment burden in primary care settings in Kerala: a cross-sectional study in Malappuram District, Kerala, India

**DOI:** 10.12688/wellcomeopenres.17674.1

**Published:** 2022-02-23

**Authors:** Sunaib Ismail, Antony Stanley, Panniyammakal Jeemon

**Affiliations:** 1 Achutha Menon Centre for Health Science Studies, Sree Chitra Tirunal Institute for Medical Sciences and Technology, Trivandrum, Kerala, 695011, India

**Keywords:** Multimorbidity, Primary Care, Kerala, India

## Abstract

**Background: **Multimorbidity or co-existence of two or more chronic conditions is common and associated with reduced quality of life and increased risk of death. We aimed to estimate the prevalence and pattern of multimorbidity in primary care settings in Kerala and the associated treatment burden, and quality of life.

**Methods: **A cross-sectional survey was conducted among 540 adult participants in Malappuram District, Kerala. A multi-stage cluster sampling method was employed. Hypertension, diabetes, chronic obstructive pulmonary disease, depression and anxiety screening were done by trained medical professionals. The remaining medical conditions were self-reported by the respondent and verified with patient held health records. The health-related quality of life [HRQoL] was measured using the EQ-5D-5L tool. The MTBQ tool was used for measuring the multimorbidity treatment burden. Logistic regression was used to identify variables associated with multi-morbidity.

**Results: **Overall, the prevalence of multi-morbidity was 39.8% (35.7 – 44.1). The prevalence of multi-morbidity among men (42.6%) was relatively higher than that in women (38.1%). Lower educational attainment, higher age group, and overweight or obesity status were independently associated with higher prevalence of multimorbidity. The most common pairs of coexisting chronic conditions reported in the study were hypertension and diabetes in males (66.7%) and females (70.8%). All domains of quality of life were impaired in individuals with multimorbidity.

**Conclusion: **Multimorbidity is a norm and affects two of five participants seeking care in primary care settings in Kerala. The social gradient in the prevalence of multimorbidity was evident with higher prevalence in individuals with low educational attainment. Multimorbidity seriously impairs quality of life and increases treatment burden. The focus of management should move beyond individual diseases, and pivot towards interventions targeting multi-morbidity management, with a specific focus for people living in lower socio-economic strata.

## Introduction

Multimorbidity is not uniformly defined in the health literature, but one of the straightforward definitions of the term is the co-existence of two or more chronic conditions in the same individual ^1, 2^. Globally, knowledge on the epidemiology of multimorbidity is suboptimal since most of the available literature highlights comorbidity around a specific disease condition of interest or comorbid pairs in association with a single index disease ^3^. Further, available data are mostly focused on populations from high-income countries.

Globally, chronic diseases account for about 41 million deaths each year, equivalent to 71% of all deaths ( World Health Organization, 2021). Out of these 41 million deaths, around 15 million occur prematurely in people aged 30–69 years. Notably, 85% of these premature deaths happen in low- and middle-income countries ^4^. Death and disability attributable to chronic conditions are largely due to deteriorating health from the coexistence of more than two conditions. Despite the variations in the number of diseases’ conditions included, and measures of multimorbidity across studies, findings suggest that approximately one in four adults have two or more chronic conditions, and half of older adults (60 years or above) have three or more chronic conditions ^1, 5– 8^.

India does not have country-level estimates on multi-morbidity from its periodic national surveys. Marked variations in prevalence exist between available research studies on multimorbidity within the country. The overall prevalence of multimorbidity in adults was 28% in a study conducted in the state of Odisha ^9^, while it was 58% among adults above the age of 50 years in a study conducted across 19 states in India ^7^. In another study with data from seven states in India ^10^ the estimated prevalence of multimorbidity in older adults (>60 years) was 30%.

In India, the epidemiological transition ratio, measured as the ratio of death and disability caused by communicable, maternal, neonatal, and nutritional diseases (CMNNDs) to those caused by non-communicable diseases (NCDs) and injuries, is lowest in the state of Kerala ^11^. Consequently, we expect a relatively higher multimorbidity burden in Kerala as compared to the rest of the country. In a recent cross-sectional study conducted in one of the southern districts in Kerala, the estimated prevalence of multimorbidity at the community level was 45% ^12^.

The state government of Kerala recently introduced localized public health projects and government-sponsored pilots (Amrutham Arogyam) in selected districts after taking cognizance of the burden and the pattern of care-seeking in primary care settings ( Arogya Keralam, 2022) The most recent primary care initiative is the ongoing phased transformation of primary health centres with high footfalls into family health centres with dedicated NCD clinics, mental health clinics, respiratory health clinics, stroke and hypertension management facilities ^13^. However, an integrated approach to managing multimorbidity is yet to be introduced in the primary care settings of Kerala.

Estimating the burden of multimorbidity at the primary care level may further help in revamping the primary care delivery design to introduce integrated patient-centred care and reduce the associated treatment burden. We aimed to estimate the prevalence and pattern of multimorbidity in primary care settings in Kerala and study associated treatment burden, and quality of life.

## Methods

### Ethics approval

Ethics clearance for the study was obtained from the Institutional Ethics Committee of Sree Chitra Tirunal Institute for Medical Sciences and Technology (SCT/IEC/1587/NOVEMBER/2020). Participants were given an information sheet and consent form in the Malayalam language. Written informed consent was obtained from all participants before enrolling them in the study. The identity of the participants was kept anonymous. Participants with abnormal values requiring immediate care were notified to the respective Medical Officer in charge of the family health centre (FHC). All locally applicable protocols for COVID-19 prevention were strictly followed during the data collection.

### Study design

The study followed a facility (family health centres of Malappuram district, Kerala) based cross-sectional design.

### Study setting

We conducted the study in the FHCs of Malappuram district from 29.12.2020 to 09.02.2021. Malappuram is the most populated district in Kerala (total population = 4,112,920) ( Census, 2011) and is situated in the northern part of the state. Based on the National Family Health Survey - 5 (NFHS-5), the sex ratio of the district was 1101. One in three women (66%) in Malappuram district reported 10 or more years of schooling, which was comparatively lower than the state average of 77%. The available studies on the prevalence of non-communicable diseases (NCD) indicate a high prevalence of comorbidities among individuals with diabetes in the district ^14^. Administratively, the district is divided into 15 health blocks. At present, the integrated services of the non-communicable diseases control programme are implemented through the 59 fully functional FHCs and they are spread across 15 blocks of the district.

### Study population

Adults (30–69) and older adults (70+) who were seeking care from the selected FHCs of Malappuram district were approached to be included in the study. The participants were residents of the district at least for the last twelve months. Those who did not give informed consent or were physically or mentally unfit to answer the questions and/or undergo clinical measurements or were pregnant/lactating mothers were excluded.

### Sample size

Based on the previously available literature, we assumed a multi-morbidity prevalence of 28% in FHC settings ^15^. The alpha and beta were kept at 0.05 and 0.20 for the sample size calculation. The sample size was calculated as 468 using the formula ((1.96) ^2^pq/d ^2^)*design effect, where ‘p’ was the anticipated prevalence, ‘q’ was ‘1-p’ and ‘d’ was the precision, which was taken as 0.05. A design effect of 1.5 was applied as the sampling was multi-stage cluster sampling. We also accounted for a 10% non-response rate and estimated the final sample size as 515.

### Sample selection

There were 59 fully functional FHCs in the Malappuram district, spread across 15 health blocks. From these 15 blocks, 6 blocks were selected using computer-generated random numbers. Further, one FHC was randomly selected from each of the selected blocks using computer-generated random numbers. Subsequently, 15 participants who met the inclusion criteria were selected consecutively from each FHC during their outpatient visit, per day. This was continued for a week (6 days) in the same FHC (i.e., 90 participants per FHC).

### Data collection

Data collection was done in the FHC setting. We used a structured interviewer-administered KoBo collect survey tool, which was prepared in English and then translated to Malayalam for data collection. Printed copies of the survey were placed in front of the participants for reference. It captured the socio-demographic factors, behavioural risk factors, and underlying medical conditions. Education was measured by capturing the highest level of formal education, and this variable was ordered into no formal schooling, primary education (Class I – Class VII), secondary education (Class VIII – Class X) and higher secondary or above.

The blood pressure (BP) was measured using a standard digital BP apparatus (Omron Blood pressure monitor-upper arm). Three readings were recorded at one-minute intervals in the non-dominant arm. We measured the random blood sugar (RBS) by using a glucometer (Onetouch Verio Flex Meter) and capillary blood. Screening for depression, anxiety and chronic obstructive pulmonary disease (COPD) were done using a validated Malayalam version of the patient health questionnaire-9 (PHQ-9) ^16^, generalized anxiety disorder-7 (GAD-7) ^17^ assessment questionnaire and COPD population screener questionnaire ^18^, respectively.

The remaining medical conditions viz. chronic kidney disease, cerebrovascular accident (CVA), coronary artery disease (CAD), heart failure, cataract, dementia, and cancer were self-reported by the respondent and verified with patient-held health records, provided by the patients. The patient held records were verified to confirm the comorbidity status. The patient held health records included consultation details (out-patient consultation form, discharge summaries), diagnostic details (specific test results), treatment details (drugs), and health insurance claims. For documenting treatment burden, we used a multi-morbidity treatment burden questionnaire (MTBQ) ^19^. We assessed the health-related quality of life (HRQoL) by using the Malayalam version of the EQ-5D-5L instrument ^20^ and derived the health status using the Indian 5-Level Version EQ-5D Value Set ^21^.

### Data collectors and quality control

A registered dental practitioner, who is undergoing post-graduate level training in public health, and two trained medical professionals were involved in data collection. The FHC medical officers and the staff nurses facilitated the data collection process. 

### Operational definitions

Multi-morbidity: The coexistence of two or more chronic conditions, from the listing of the following 12 conditions (hypertension, diabetes mellitus, depression, anxiety, COPD, chronic kidney disease, coronary artery disease (CAD), cerebrovascular accident (CVA), heart failure, cataract, dementia and cancer), in the same individual.

Hypertension: As per Joint National Committee-7 guidelines ^22^.

Diabetes mellitus: A random capillary blood glucose value above 140mg/dl.

Depression: PHQ-9 score of 10 or above ^16^.

Anxiety: GAD-7 score of 10 or above ^17^.

Health-related quality of life (HRQoL): EQ-5D-5L ^20, 21^

Multimorbidity Treatment Burden: The MTBQ is a 10-item measure of treatment burden for patients with multimorbidity ^19^. The study participants were categorized into high, medium, low or no treatment burden groups based on global MTBQ scores (≥ 22=high, 10–22=medium, <10=low or no treatment burden).

### Data management and data analysis

The data collected via KOBO collect were downloaded and checked for incongruencies and missing data. Data cleaning was carried out using Microsoft Excel 2019. It was then imported to SPSS version 25 for analysis. Descriptive statistics were used to describe the study population by summarising the distribution of relevant variables. Continuous variables were presented as mean and standard deviation and categorical variables as proportions and percentages. One-way ANOVA was used to compare the differences in means of HRQoL and multimorbidity treatment burden across groups based on the number of chronic conditions. A backward stepwise logistic regression model was used to identify socio-demographic factors that were independently associated with multi-morbidity and generated odds ratio with its 95% confidence interval. The level of statistical significance was set at a p-value of less than 0.05.

## Results

### Socio-demographic characteristics of the respondents

Overall, 540 participants completed the interview, out of which 324 (60%) were women. No participants had missing data. The mean age of the study participants was 56.7 (12.0) years ( Table 1). One in four participants reported no formal schooling (26%), and more than half of the respondents were in the unemployed category (52%). Three in five men (64%) and women (61%) were either overweight (BMI = 23.0 - 24.9) or obese (BMI >25.0). Alcohol and tobacco use were reported only among men.

**Table 1.** Sample characteristics among men and women.

Variable	Total (N=540)	Men (N=216)	Women (N=324)
Age (in years), mean (SD *)	56.7 (12.0)	57.7 (12.6)	56.0 (11.6)
**Age group, n (%)**			
30–49	145 (26.9%)	57 (26.4%)	88 (27.2%)
50–69	308 (57.0%)	112 (51.9%)	196 (60.5%)
70+	87 (16.1%)	47 (21.8%)	40 (12.3%)
**Educational status, n (%)**			
No formal schooling	139 (25.7%)	50 (23.1%)	89 (27.5%)
Primary (Class I – Class VII)	231 (42.8%)	87 (40.3%)	144 (44.4%)
Secondary (Class VIII – Class X)	123 (22.8%)	56 (25.9%)	67 (20.7%)
Higher secondary or above	47 (8.7%)	23 (10.6%)	24 (7.4%)
**Work Status, n (%)**			
Government employee	18 (3.3%)	7 (3.2%)	11 (3.4%)
Skilled Labourer	27 (5.0%)	14 (6.5%)	13 (4.0%)
Unskilled/Manual Labourer	118 (21.9%)	60 (27.8%)	58 (17.9%)
Homemaker	95 (17.6%)	9 (4.2%)	86 (26.5%)
Unemployed	282 (52.2%)	126 (58.3%)	156 (48.1%)
**Tobacco and alcohol use, n (%)**			
Smoking tobacco (ever use)	57 (10.6%)	57 (26.8%)	0
Chewable tobacco (ever use)	14 (2.6%)	14 (6.4%)	0
Any form of tobacco (ever use)	71 (13.1%)	71 (32.8%)	0
Alcohol consumption (ever use)	38 (7%)	38 (17.5%)	0
Body Mass Index, mean (SD)	23.8 (2.0)	23.7 (0.4)	23.8 (2.0)
Overweight, n (%)	163 (30.2%)	67 (31.0%)	96 (29.6%)
Obese, n (%)	173 (32.0%)	71 (32.9%)	102 (31.5%)

* SD = Standard deviation

### Prevalence and patterns of multi-morbidity

**Figure 1.**
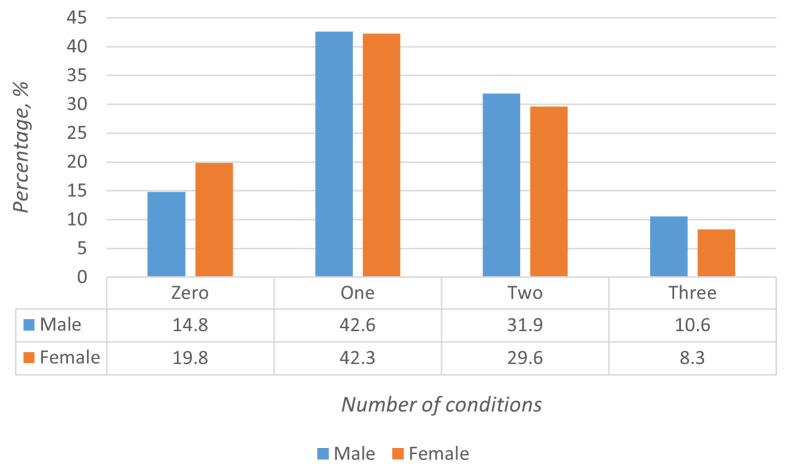
Multi-morbidity prevalence among males and females.

**Figure 2.**
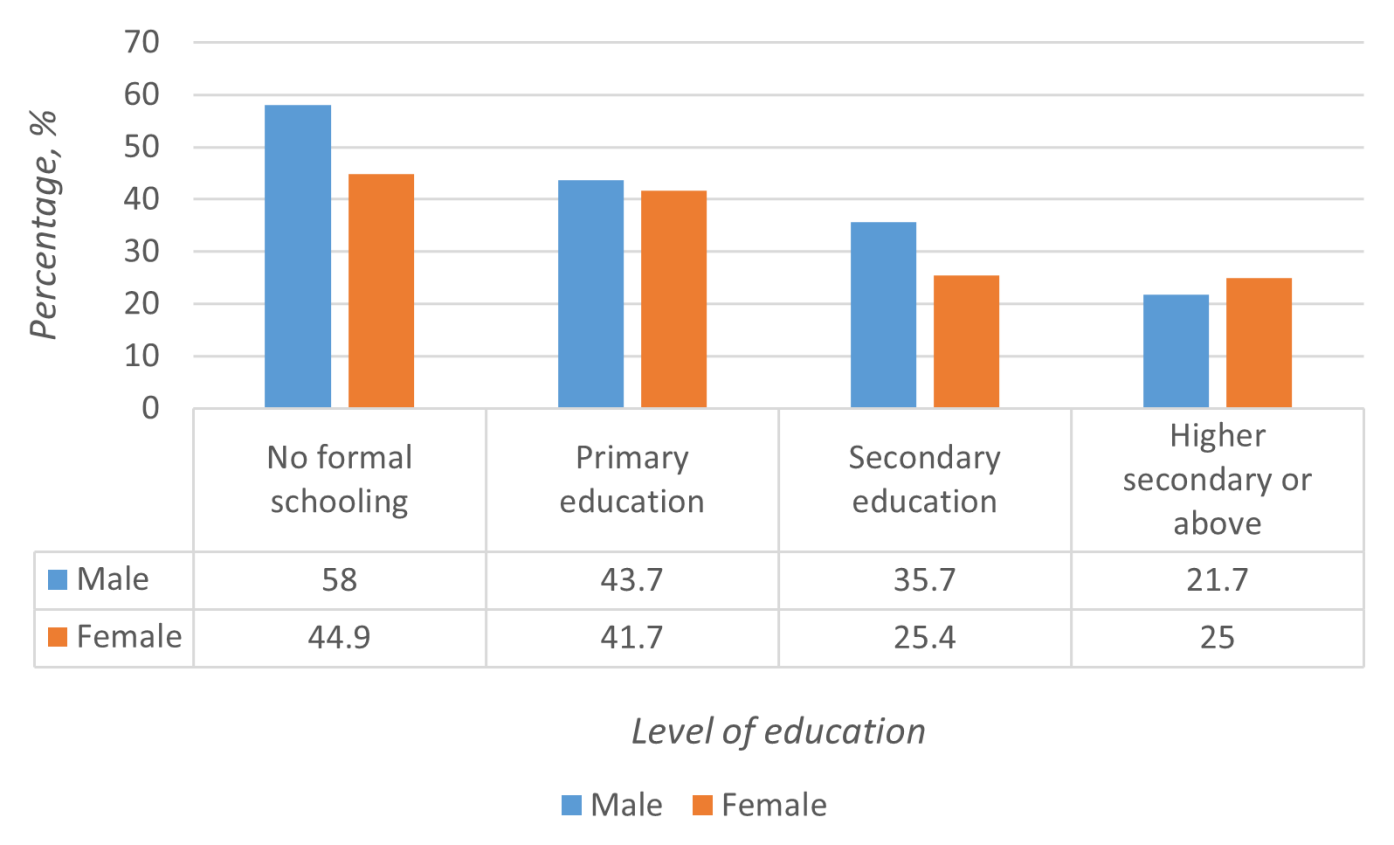
Prevalence of multi-morbidity based on education status.

***Prevalence of multi-morbidity*.** Among the study population, hypertension was the most prevalent morbidity (68.1%) followed by diabetes (37.2%) in both sexes ( Table 2). One condition was reported by 229 (42.4%) participants, while 165 (30.6%) presented with two and 50 (9.3%) participants reported three or more coexisting conditions. Overall, 215 participants (39.8%) reported multi-morbidity ( Figure 1). The prevalence of multi-morbidity increased with age and decreased with an increase in the educational level of the study participants ( Figure 2). Age-wise, the highest prevalence of multi-morbidity was in the 50–69 year age group. However, multi-morbidity was a notable problem even in the younger (30–49 years) population (19.3%). The prevalence of multi-morbidity among men (42.6%) was relatively higher than that in women (38.1%).

**Table 2.** Prevalence of individual conditions.

Conditions	Total (N=540)	Men (N=216)	Women (N=324)
Hypertension, n (%)	368 (68.1%)	146 (67.5%)	222 (68.5%)
Diabetes, n (%)	201 (37.2%)	82 (37.9%)	119 (36.7%)
COPD/Asthma, n (%)	32 (5.9%)	16 (7.4%)	16 (4.9%)
Renal diseases, n (%)	27 (5.0%)	12 (5.5%)	15 (4.6%)
CVA ^❖^, CAD ^♦^, or Heart failure	27 (5.0%)	12 (5.5%)	15 (4.6%)
Cataract, n (%)	11 (2.0%)	6 (2.8%)	5 (1.5%)
Dementia, n (%)	7 (1.3%)	4 (1.8%)	3 (0.9%)
Cancer, n (%)	3 (0.5%)	1 (0.5%)	2 (0.6%)
Depression, n (%)	31 (5.7%)	9 (4.2%%)	22 (6.8%)
Anxiety, n (%)	15 (2.8%)	6 (2.8%)	9 (2.8%)

* Depression was defined as a PHQ-9 score of 10 or above # Anxiety was defined as a GAD-7 score of 10 or above ❖ Cerebrovascular accident ♦ coronary artery disease

In the multivariate analyses ( Table 3), multi-morbidity was associated with the educational status, age group, and obesity. The propensity for multi-morbidity was higher in individuals in the higher age group, for 50–69 years (adjusted odds ratio, aOR=2.9; 95% CI: 1.5-5.4, p=0.01) and 70+ years (aOR=3.0; 95% CI: 1.3-6.6, p=0.01) as compared to younger age groups. Similarly, propensity for multi-morbidity was higher in overweight/obese (aOR=36.3; 95% CI: 20.7 – 63.7), p<0.001) individuals as compared to normal-weight individuals. Lower educational attainment was associated with multimorbidity. For example, the participants who had attained secondary education (aOR=0.43; 95% CI: 0.21-0.89, p=0.02) or higher secondary education or above (aOR=0.30; 95% CI: 0.10-0.89, p=0.03) had lower odds of multi-morbidity compared to individuals with no formal schooling.

**Table 3.** Association between socio-demographic characteristics and multimorbidity (MM).

Variables	MM present (N = 215)	MM absent (N = 325)	Adjusted OR, 95% CI	P value *
**Sex,** n (%)				
Men	92 (42.6%)	124 (57.4%)	**Reference group**	
Women	123 (38.1%)	201 (62.0%)	0.71 (0.44 – 1.17)	0.28
**Age group,** n (%)				
30–49 years	28 (19.4%)	117 (80.7%)	**Reference group**	
50–69 years	146 (47.4%)	162 (52.6%)	2.87 (1.52 – 5.42)	0.001
70+ years	41 (47.1%)	46 (52.9%)	2.97 (1.32 – 6.63)	0.008
**Educational status,** n (%)				
No formal schooling	69 (49.6%)	70 (50.4%)	**Reference group**	
Primary (Class I – VII)	98 (42.4%)	133 (57.6%)	0.80 (0.45 – 1.44)	0.46
Secondary (Class VIII – X)	37 (30.1%)	86 (69.9%)	0.43 (0.21 – 0.89)	0.02
Higher secondary or above	11 (23.4%)	36 (76.6%)	0.30 (0.10 – 0.89)	0.03
**Obesity,** n (%)				
Normal	55 (16.2%)	284 (83.8%)	**Reference group**	
Overweight/ Obese	160 (79.6%)	41 (20.4%)	36.27 (20.67 – 63.65)	<0.001

* Only variables that showed statistical significance (p<0.2) in unadjusted analyses were entered into the adjusted multivariate analysis. The independent variables are sex, education status, age group and BMI category. CI: confidence interval, OR: Odds Ratio

***Patterns of multi-morbidity*.** The most common pairs of coexisting chronic conditions ( Table 4) reported in the study were hypertension and diabetes in males (66.7%) and females (70.8%), followed by hypertension and COPD/asthma in males (8.7%) and hypertension and depression in females (8.3%). The most common triad in females was diabetes, hypertension, and COPD/asthma (27.8%), while in males it was diabetes, hypertension, and heart disease (21.0%).

**Table 4.** Top three dyads and triads of multi-morbidity.

Dyads, n (%)	Total (N = 165)	Men (N = 69)	Women (N = 96)
Hypertension – Diabetes	114 (69.1%)	46 (66.7%)	68 (70.8%)
Hypertension – COPD/Asthma	12 (7.3%)	6 (8.7%)	6 (6.2%)
Hypertension – Depression	11 (6.7%)	3 (4.3%)	8 (8.3%)
Triads, n (%)	Total (N = 50)	Men (N = 23)	Women (N = 27)
Hypertension – Diabetes – COPD/Asthma	7 (18.9%)	2 (10.5%)	5 (27.8%)
Hypertension – Diabetes – Heart disease	7 (18.9%)	4 (21.0%)	3 (16.7%)
Hypertension – Anxiety – Depression	6 (16.2%)	3 (15.8%)	3 (16.7%)

# The complete list of dyads and triads are attached as annexures in the supporting information section.

### Association of multi-morbidity with quality of life and multi-morbidity treatment burden

**Figure 3.**
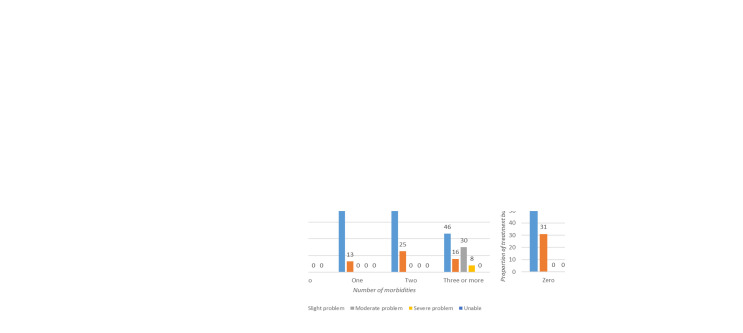
EQ-5D-5L domains, multimorbidity treatment burden and multimorbidity.

***Quality of life and multi-morbidity treatment burden*.** The HRQoL derived from the EQ-5D-5L deteriorated with an increase in the number of chronic conditions ( Figure 3). Based on both HRQoL and the VAS score, individuals with multiple chronic conditions reported poor quality of life as compared to individuals with no or single chronic conditions ( Table 5). All domains of quality of life such as mobility, usual care activities, depression/anxiety, self-care, pain/discomfort were impaired in individuals with multiple chronic conditions ( Figure 3). The treatment burden associated with multi-morbidity showed a positive linear association with the number of co-existing chronic conditions ( Table 5). Medium to high treatment burden was mostly noted in individuals with two and three or more chronic conditions ( Figure 3) as compared to individuals with no or one chronic condition.

**Table 5.** Quality of life and treatment burden according to number of chronic conditions.

Characteristic	Number of multimorbid conditions				P-Value *
	Zero (N = 96)	One (N = 229)	Two (N = 165)	Three or more (N = 50)	
Quality of life, mean (SD)	1 (0.0)	0.7 (0.1)	0.5 (0.1)	0.2 (0.2)	<0.001
VAS scores, mean (SD)	87.1 (6.3)	89.3 (7.2)	71.1 (3.7)	58.6 (7.9)	<0.001
Multi-morbidity treatment burden, mean (SD)	1.6 (3.1)	9.7 (3.8)	19.8 (5.8)	23.3 (5.6)	<0.001

* P-value calculated using One way ANOVA. SD: Standard deviation

## Discussion

We demonstrate that multimorbidity is almost a norm and prevalent in two in five participants seeking care from FHCs in Malappuram district, Kerala. The inverse relationship between educational status and multimorbidity, with the burden largely concentrated among the low education group, clearly highlights the reversal of social gradient in the prevalence of chronic disease conditions. Multimorbidity is a serious public health problem in the primary care settings in Kerala as it impairs the quality of life. The high treatment burden associated with multimorbidity could be partially attributable to the number of chronic conditions and partially to the lack of integrated care for the management of multimorbidity in the primary care system.

### Prevalence and patterns of multi-morbidity

Lack of uniformity in the methods of data collection, study instruments, and the number of conditions covered across different studies limits the opportunity for direct comparison of the prevalence estimates with other studies. In a relatively comparable primary care-based study done in Odisha state, the prevalence of multi-morbidity was around 28% ^23^. The substantially higher prevalence of multimorbidity in our study is probably due to the advanced epidemiological transition state of Kerala as compared to Odisha ^11^. It is now understood that areas in advanced epidemiological transition have a higher frequency of multi-morbidity ^11^. Men reported a relatively higher prevalence of multi-morbidity than women among the set of morbidities studied, which contradicts previous research ^24, 25^. However, only men reported smoking and drinking in our study, which may have contributed to the increased frequency of multimorbidity among men. Further, exclusion of pregnant and lactating women from the study may have resulted in the underestimation of multimorbidity as conditions like depression is more common in antenatal and post-partum period ^26^.

### Multi-morbidity status assessment with education status of the participants

In recent studies from lower-middle-income countries, increasing levels of deprivation are consistently associated with a higher risk of multimorbidity ^27^. In our study, the propensity for multi-morbidity was lower in the well-educated group as compared to individuals with poor educational attainment. Education is often considered a good proxy measure of a family's socioeconomic status ^28^. Our findings highlight the importance of the social determinants of multimorbidity and call for integrated management programmes that incorporate strategies to address social determinants along with the medical needs. The vicious cycle of poor socio-economic status and high burden of NCDs is well established and it may further propel the burden of multimorbidity in the disadvantaged social groups.

### Multi-morbidity prevalence assessment in subgroups and their implications

The age group of 50–69 years reported the highest prevalence of multimorbidity, which matched with findings from other studies ^29^. Even though five out of ten participants in the older age groups (50–69 years and 70/70+ years) reported multi-morbidity, it was also prevalent in two of five in the younger age groups (30–49 years). Multimorbidity in the young adult age group may result in disproportionately high productivity loss as compared to other groups. Further, the progression of multimorbidity with more conditions later in life may adversely impact the health and productivity of these individuals. However, it is not clear how multimorbidity progresses in individuals and we need to generate data from well-designed cohort studies or registries of patients with multimorbidity on a regular follow-up to understand the progression. Integrated management with a focus on prevention may help to reduce the burden of multimorbidity in primary care settings. Novel models of prevention and control of multimorbidity need to be developed and evaluated before the wider adoption of these strategies in primary care settings. From the policy perspective, the identification of groups vulnerable to multimorbidity will help in the selection of preventive public health interventions to reduce the multimorbidity burden in high-risk groups.

### Multi-morbidity, quality of life and treatment burden and their implications

Diabetes and hypertension were the most common coexisting chronic illnesses in both males and females in our study. This is consistent with the earlier multi-morbidity studies from India ^30^. Hypertension was the associated comorbidity in the most prevalent dyads and triads. In our study, multimorbidity in any combination impaired the quality of life and increased the treatment burden. A comparison with existing literature shows that similar findings are consistently seen across the world ^31, 32^. It highlights that multimorbidity is a serious universal public health challenge and the lack of recognition of multimorbidity may potentially increase the disability associated with chronic conditions. New intervention models that could improve the quality of life of patients with multimorbidity need to be developed and evaluated in primary care settings.

### Strength and limitations

The major strength of our study was the high response rate. In addition, we actively screened several chronic conditions, rather than relying on self-reported disease conditions. A medically qualified team conducted the screening, and the self-reported information was also cross-checked for accuracy. The research team explored the HRQoL using the EQ-5D-5L value set for India, which helps in overcoming the cultural and geographical biases associated with using value sets of other countries. The major limitation of the study was that the severity of chronic illnesses was not taken into consideration in the multi-morbidity assessment. Further, this is a cross-sectional study, and therefore the causality of the association cannot be established.

## Conclusion

Multi-morbidity is a norm and affects two out of five participants seeking care in primary care settings in Kerala. Hypertension and diabetes are the most common co-existing conditions. The social gradient in the prevalence of multimorbidity was evident with higher prevalence in individuals with low educational attainment. Multimorbidity seriously impairs quality of life and increases treatment burden to the affected individuals. The focus of management should move beyond individual diseases, and pivot towards interventions targeting multi-morbidity management, with a specific focus for people living in lower socio-economic strata and younger age groups to improve their quality of life.

## Data availability

### Underlying data

figshare: Prevalence of multimorbidity, treatment burden and related quality of life in primary care settings in Kerala: A cross-sectional study in Malappuram, Kerala. https://doi.org/10.6084/m9.figshare.17277167 ^33^

 This project contains the following underlying data:

- Data_Multimorbidiy.xlsx (raw data)

figshare: Dataset for the study. https://doi.org/10.6084/m9.figshare.17277167.v1 ^34^

This project contains the following underlying data:

- Codes_Multimorbidiy (1).xlsx (data key)

Data are available under the terms of the Creative Commons Attribution 4.0 International license (CC-BY 4.0).

### Extended data

figshare: Interview schedule in English with codes. https://doi.org/10.6084/m9.figshare.17274791.v1 ^35^

figshare: Interview schedule in Malayalam with codes. https://doi.org/10.6084/m9.figshare.17274938.v1 ^36^

figshare: Full list of dyads & triads. https://doi.org/10.6084/m9.figshare.17283761.v1 ^37^

figshare: Consent form. https://doi.org/10.6084/m9.figshare.18586052.v2 ^38^

Data are available under the terms of the Creative Commons Attribution 4.0 International license (CC-BY 4.0).
