# Supplementary material for: Prevalence of multimorbidity and associated treatment burden in primary care settings in Kerala: a cross-sectional study in Malappuram District, Kerala, India
Source: Wellcome Open Res. 2022 May 4;7:67. Originally published 2022 Feb 23. [Version 2] doi: 10.12688/wellcomeopenres.17674.2 (PMC9086527; doi:10.12688/wellcomeopenres.17674.2)
Supplement: Peer review history

# Peer review: Reviewer response for version 2

**DOI:** 10.21956/wellcomeopenres.19791.r50295

**Published:** 2022-05-09

**Authors:** Filipe Prazeres

**Affiliations:** 1 Faculty of Health Sciences, University of Beira Interior, Covilhã, Portugal

I wish to thank the authors for their responses to my questions and I have no further comments to make.

Is the work clearly and accurately presented and does it cite the current literature?

Yes

If applicable, is the statistical analysis and its interpretation appropriate?

Yes

Are all the source data underlying the results available to ensure full reproducibility?

Yes

Is the study design appropriate and is the work technically sound?

Partly

Are the conclusions drawn adequately supported by the results?

Yes

Are sufficient details of methods and analysis provided to allow replication by others?

Partly

Reviewer Expertise:

multimorbidity; chronic diseases; family medicine; primary care; quality of life; geriatrics; public health

I confirm that I have read this submission and believe that I have an appropriate level of expertise to confirm that it is of an acceptable scientific standard.

# Peer review: Reviewer response for version 1

**DOI:** 10.21956/wellcomeopenres.19552.r49832

**Published:** 2022-04-12

**Authors:** Filipe Prazeres

**Affiliations:** 1 Faculty of Health Sciences, University of Beira Interior, Covilhã, Portugal

Thank you for allowing me to read this interesting article regarding the prevalence of multimorbidity in India. As stated by the authors, most of the available data are mostly focused on populations from high-income countries, and so this data is of importance to add to the body of knowledge. Nonetheless, I have some comments to improve the manuscript:

- Since the year 2018 multimorbidity is a MeSH term ( https://www.ncbi.nlm.nih.gov/mesh/?term=multimorbidity). Please use multimorbidity instead of multi-morbidity throughout the paper.

**Methods:**

- Can the authors please explain why did they use the following criteria to diagnose Diabetes mellitus: A random capillary blood glucose value above 140mg/dl? The Criteria for the Screening and Diagnosis of Prediabetes and Diabetes ADA 2022 ^1^ when using Random plasma glucose is ≥200 mg/dL (11.1 mmol/L) 
- Why did the authors consider adults only the individuals  >30years of age?
- 12 conditions were chosen by the authors to define multimorbidity. Can the authors please explain their choice criteria?
- I also agree with Dr. Soumya Gopakumar that the authors need to clarify the definition of treatment burden. Sometimes the reader does not understand what the authors mean when they use this term.

**Results**

- Figure 1 - The number of conditions stops at 3. There were no individuals with more than 3 co-occurring disorders?
- Figure 3 is in a small format and because of that, it is difficult to read.

Is the work clearly and accurately presented and does it cite the current literature?

Yes

If applicable, is the statistical analysis and its interpretation appropriate?

Yes

Are all the source data underlying the results available to ensure full reproducibility?

Yes

Is the study design appropriate and is the work technically sound?

Partly

Are the conclusions drawn adequately supported by the results?

Yes

Are sufficient details of methods and analysis provided to allow replication by others?

Partly

Reviewer Expertise:

multimorbidity; chronic diseases; family medicine; primary care; quality of life; geriatrics; public health

I confirm that I have read this submission and believe that I have an appropriate level of expertise to confirm that it is of an acceptable scientific standard, however I have significant reservations, as outlined above.

## References

[1] : Standards of Medical Care in Diabetes-2022 Abridged for Primary Care Providers. *Clin Diabetes* .2022;40(1) : 10.2337/cd22-as01 10-38 10.2337/cd22-as01 35221470PMC8865785

# Response

**Published:** 2022-04-22

**Authors:** Panniyammakal Jeemon

**Affiliations:** Sree Chitra Tirunal Institute for Medical Sciences and Technology, Trivandrum, India

Thank you very much for the comments. We have changed 'multi-morbidity' to 'multimorbidity' throughout the manuscript.

 We used the random glucose criteria to identify individuals with diabetes based on studies conducted in the Indian context (Susairaj P, Snehalatha C, Raghavan A, Nanditha A, Vinitha R, Satheesh K, Johnston DG, Wareham NJ, Ramachandran A. Cut-off Value of Random Blood Glucose among Asian Indians for Preliminary Screening of Persons with Prediabetes and

 Undetected Type 2 Diabetes Defined by the Glycosylated Haemoglobin Criteria. J

 Diabetes Clin Res. 2019;1(2):53-58, and Somannavar S, Ganesan A, Deepa M, Datta M, Mohan V. Random capillary blood glucose cut points for diabetes and pre-diabetes derived from community-based

 opportunistic screening in India. Diabetes Care. 2009 Apr;32(4):641-3.).

 We included adults >30years of age in our study based on the Revised operational guidelines of the National Program for Prevention and Control of Cancer, Diabetes, CVD and Stroke (( Operational Guidelines of NPCDCS (Revised - 2013-17)_1.pdf (mohfw.gov.in)).

 The twelve conditions that we have included in our study for multimorbidity assessment are selected from the Academy of Medical Sciences (UK) report on multimorbidity.

 The treatment burden is assessed based on the Multimorbidity Treatment Burden Questionnaire (MTBQ). It is a validated 10-item questionnaire, which covers multiple domains of treatment burden such as coordinating healthcare appointments, taking multiple medicines, self-monitoring their health and making lifestyle changes.

 The last category in Fig 1 was three or more conditions and we have corrected the error in the revised version. We have divided the Fig 3 into Fig 3A-3F and provided them separately.

# Peer review: Reviewer response for version 1

**DOI:** 10.21956/wellcomeopenres.19552.r49022

**Published:** 2022-03-14

**Authors:** Soumya Gopakumar

**Affiliations:** 1 Department of Community Medicine, Government Medical College Thiruvananthapuram, Thiruvananthapuram, Kerala, India

This is a well written article with suitable design. The authors have beautifully described the screening procedures used in the study which can be replicated. However I would like to point out that, the study was done in a single district in Kerala. The study subjects included patients attending family health centres only. In a state like Kerala, people depend on private health care providers also. In the present study multi-morbidity was lower in the well-educated group. Education alone may not be good proxy measure of a family's socioeconomic status especially in a district like Malappuram as many family members may be living abroad and adds to the family income. They may seek health in a private hospital. In sample size calculation, precision to be specified as 5 % absolute precision. There is a lack of clarity in the term treatment burden.

Is the work clearly and accurately presented and does it cite the current literature?

Yes

If applicable, is the statistical analysis and its interpretation appropriate?

Yes

Are all the source data underlying the results available to ensure full reproducibility?

Yes

Is the study design appropriate and is the work technically sound?

Yes

Are the conclusions drawn adequately supported by the results?

Partly

Are sufficient details of methods and analysis provided to allow replication by others?

Yes

Reviewer Expertise:

Public health, research methods

I confirm that I have read this submission and believe that I have an appropriate level of expertise to confirm that it is of an acceptable scientific standard.

# Response

**Published:** 2022-04-22

**Authors:** Panniyammakal Jeemon

**Affiliations:** Sree Chitra Tirunal Institute for Medical Sciences and Technology, Trivandrum, India

Thank you very much for the positive comments. We have included the name of the district in the study title itself. We agree that we have not considered patients seeking primary care from private facilities in Kerala. We accept it as a limitation of the study. Educational level has been widely accepted as a proxy for socio-economic status (Reddy KS, Prabhakaran D, Jeemon P, Thankappan KR, Joshi P, Chaturvedi V, Ramakrishnan L, Ahmed F. Educational status and cardiovascular risk profile in Indians. Proc Natl Acad Sci U S A. 2007 Oct 9;104(41):16263-8.). It is also difficult to assess the income due to reporting errors. The treatment burden is estimated using a validated questionnaire, which assesses multiple domains of treatment burden such as coordinating healthcare appointments, taking multiple medicines, self-monitoring health and making lifestyle changes ( https://www.bristol.ac.uk/primaryhealthcare/resources/mtbq/).
